# Middle Eastern Rhinoplasty: Update

**DOI:** 10.1097/GOX.0000000000001984

**Published:** 2018-12-18

**Authors:** Rod J. Rohrich, Raja Mohan

**Affiliations:** From the Dallas Plastic Surgery Institute, Dallas, Tex.

## Abstract

Middle Eastern rhinoplasty requires especially precise preoperative planning to achieve a successful result. Among all aesthetic surgery procedures, there is a higher demand for rhinoplasty among Middle Eastern cultures. The key is to maintain the ethnicity of the patients while meeting their goals. In this brief overview, common features in Middle Eastern patients and Middle Eastern rhinoplasty techniques in men and women are highlighted.

## INTRODUCTION

Middle Eastern rhinoplasty is a topic of special importance within the broader category of ethnic rhinoplasty. Among all aesthetic surgeries, rhinoplasty is the one most commonly performed in this population. Tehran in Iran has been termed the “rhinoplasty capital of the world” and the rate of rhinoplasty is 7 times more than that of United States.^1^ The term Middle Eastern is quite broad and includes individuals: (1) Gulf countries (Iran, Saudi Arabia, Kuwait, Qatar, Oman, Bahrain, United Arab Emirates, Yemen); (2) North Africa (Egypt, Libya, Algeria, Morocco); (3) various other countries (Lebanon, Afghanistan, Syria, Turkey, Greece, Armenia).^2–4^ Although these regions encompass most Middle Eastern patients, there are salient differences between patients. In one review, patients from non-Gulf countries preferred more tip projection and less of a dorsal hump compared with other regions.^5^

In ethnic rhinoplasty, the key is to avoid changing one’s ethnic identity or appearance by making radical changes.^4^ Attempting to perform a rhinoplasty meant for a lower Fitzpatrick grade on patients with broader features and thicker skin may create an oversculpted appearance. Middle Eastern patients typically have reasonable requests with very high expectations.^6^ Despite their expectations, an outcomes study reported higher quality of life in Middle Eastern patients who underwent rhinoplasty.^7,8^ This surgery can be very rewarding for these patients.

## NASAL ANALYSIS

**Fig. 1.**
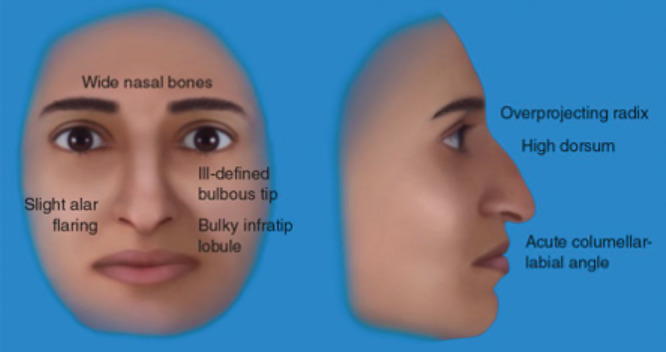
The nasal appearance in a Middle Eastern nose demonstrates the following: high dorsum, a dependent or ill-defined nasal tip and a thick soft-tissue envelope. (*From Dallas Rhinoplasty 3^rd^ Edition, Eds: Rod J. Rohrich, William P. Adams Jr., Jamil Ahmad, and Jack P. Gunter. 2014, Boca Raton: CRC Press. Reprinted with Permission*).

**Fig. 2.**
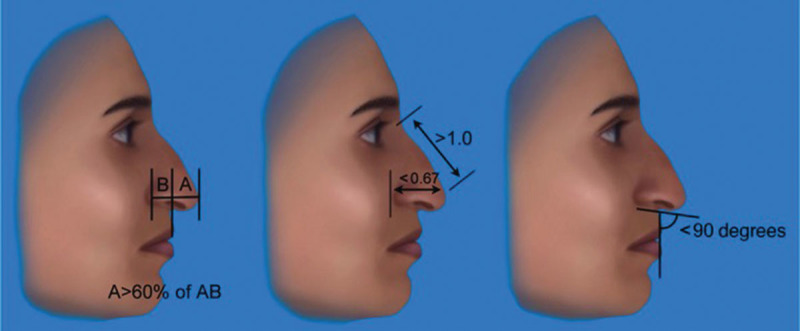
On lateral view, A (defined as the segment from the upper lip to the tip) is commonly greater than 60% of the distance from the alar-facial crease to the tip (B). Nasal length is often much longer relative to the tip projection. A plunging tip can give the appearance of poor nasal projection. The nasolabial and columellar-labial angles are frequently less than 90 degrees in a plunging tip. *(From Dallas Rhinoplasty 3^rd^ Edition, Eds: Rod J. Rohrich, William P. Adams Jr., Jamil Ahmad, and Jack P. Gunter. 2014, Boca Raton: CRC Press. Reprinted with Permission*).

Among rhinoplasty surgeons and in the literature, it is traditionally believed that people of Middle Eastern descent have the following nasal characteristics: thick skin, a dorsal hump, weak lower lateral cartilages, under-projected tip, acute nasolabial angle, and tip asymmetries^1–3,9–11^ (Fig. 1). On the profile view, people of Middle Eastern descent may have a dorsal hump and inadequate tip projection and the basal view demonstrates possible tip asymmetry or deviation^2^ (Fig. 2). The key features to remember are the presence of thick skin, weak lower lateral cartilages or framework, and deficient septal cartilage.

A review of the characteristics identified in the literature (Table 1), and a complete list of characteristics of the Middle Eastern nose are shown (Table 2). The features that are uncommon in this population are also listed (Table 3). Although the features in Table 2 are quite common within these populations, they are generalizations and do not apply to all Middle Eastern patients. Each patient requires an individualized and systematic analysis.

**Table 1.**
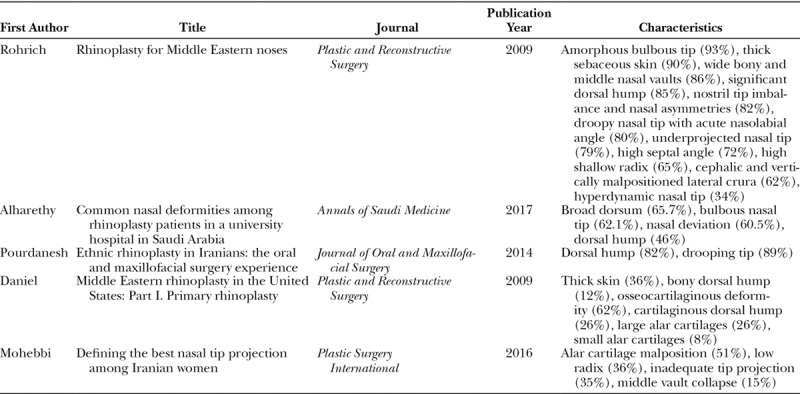
Review of Characteristics Identified in the Middle Eastern Nose

**Table 2.**
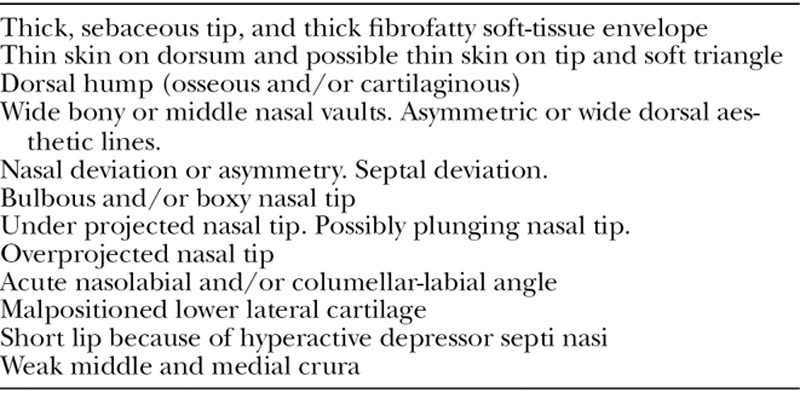
Characteristics of the Middle Eastern Nose

**Table 3.**
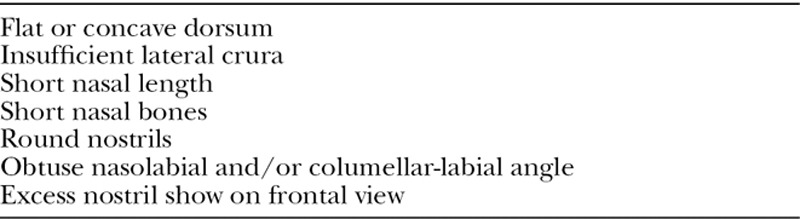
Features That Are Rare in the Middle Eastern Nose

We have reviewed how to perform a thorough nasofacial analysis in patients desiring rhinoplasty.^12^ In the nasal analysis of Middle Eastern patients, the following characteristics must be thoroughly assessed: Fitzpatrick skin type, skin thickness, dorsal position, nasal length, orientation and strength of lateral crura, amount of nasal deviation, alar flaring/sill excess, nostril tip imbalance, alar base position, columellar and medial crura length, and the presence of a hyper-dynamic tip.

To assess tip projection, on lateral view, the segment from the upper lip to the tip should be at least 60% of the total length (from facial crease to the tip) (Fig. 2). Nasal length in these patients is typically long, especially with a plunging tip. The nasolabial and columellar-labial angle are acute in patients with a plunging tip. The assessment of the tip is more complicated in these patients because they may present with tip asymmetries and abnormalities associated with projection and rotation. Sometimes what is perceived as an under-projected tip may be an under-rotated tip. Causes of tip ptosis include lower lateral cartilage malposition, overdevelopment of the upper lateral cartilages, thick skin, and a prominent anterior septal angle.^1^

## SKIN AND SOFT TISSUE

**Fig. 3.**
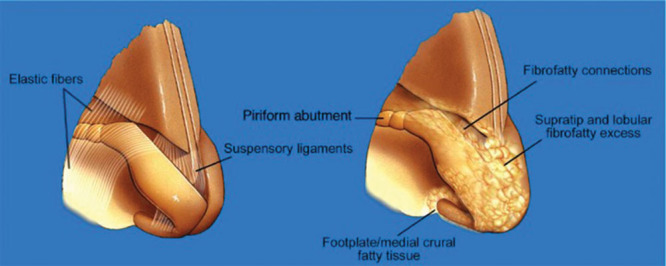
The abundance of intercartilaginous fibrofatty tissue can be responsible for the decreased stability of the cartilaginous framework. The fibroligamentous nasal attachments can be weakened by the fibrofatty tissue. Weak tip cartilages make the nasal tip and lobule more compressible. *(From Dallas Rhinoplasty 3^rd^ Edition, Eds: Rod J. Rohrich, William P. Adams Jr., Jamil Ahmad, and Jack P. Gunter. 2014, Boca Raton: CRC Press. Reprinted with Permission*).

Middle Eastern patients usually have Fitzpatrick type III to V skin although there are exceptions. The skin is more sebaceous at the lobule and along the alar rims. Some of the thickness at the tip is secondary to a large amount of fibrofatty soft tissue between the medial crura. The ligamentous attachments could be weakened by the excess fibrofatty tissue.^13^ When the lobule is larger and thicker, it has a very soft and spongy feel with weak lower lateral cartilages (Fig. 3). The medial and middle crura tend to be insufficient and caudally displaced because of their inherent weakness. In patients with thick nasal skin and fibrofatty tissue, the soft tissue can be thinned and the tip complex can be supported with visible grafting and suturing techniques.^14,15^ Thicker skin makes it harder to visualize the effects of a rhinoplasty because the tissue does not contract, and its healing is unpredictable.

In patients with thin skin, no defatting should be performed and after any reduction maneuvers, the bony vault and middle vault should be assessed for any irregularities. Any tip grafts placed should be of lower profile to prevent palpability and visibility. The key in Middle Eastern rhinoplasty is creating a strong internal framework that provides tip support and achieves the goals of various rhinoplasty techniques.

## DORSUM

The nasal dorsum is wide and more prominent in Middle Eastern patients. A normal radix is located between the upper lid margin and the supratarsal fold. The ratio of the distance from the cornea to the radix should be 0.28 when compared with the nasal length.^16^ Some Middle Eastern patients may have an overprojected radix and a lower radix is acceptable in some patients. Component dorsal reduction is performed for any osseocartilaginous hump.^15,17^ The goal is not to overreduce the bony or cartilaginous dorsum resulting in a sloped or scooped out appearance. Reduction of the radix can also be performed to set a dorsal height set-point.^18^ Dorsal height needs to be congruent with projection of the radix. To narrow a wide dorsum, close an open-roof deformity, or create more symmetric dorsal aesthetic lines, osteotomies are performed using a low-to-low percutaneous technique.^19^ Any septal or nasal deviation should be corrected with septal reconstruction and harvested septum can be used for grafting.^15^ We do not routinely use spreader grafts in primary rhinoplasty and recommend upper lateral cartilage tension spanning sutures^20^ or auto-spreader flaps.

## TIP REFINEMENT

Tip support is dependent on 5 different factors including the medial crural attachments, the scroll area, attachment of anterior septal angle to the tip, lateral crura, and the septum.^5^ In the scroll area, there is an extended lateral junction that is unique to Middle Eastern patients.^14^ Tip modification is common among Middle Eastern patients since these patients can present with deformities associated with projection, asymmetry, rotation, or bulbosity.

Invisible grafts and suturing techniques may not show any visible changes if the patient has a thick soft-tissue envelope. A graduated approach of suturing utilizing medial crural, medial crural-septal, trans-domal, and intra-domal sutures allows one to strengthen the tip and control the projection and rotation.^21^ In select patients who have short and weak middle and medial crura, they may need a columellar strut to lengthen and strengthen the lower lateral cartilages.^22^ Tip ptosis or a plunging tip can be addressed by rotating the tip upward using suturing techniques. Tip projection is obtained by strengthening the lower lateral cartilages using suturing and grafting techniques.^15,23^ Other techniques include lateral crural steal, lateral crural overlap, and tongue-in-groove to increase tip projection and add strength.^10^ Visible grafts are generally needed to provide adequate tip support and creation of a strong internal framework.

The management of bulbous and boxy tips has been described by Rohrich and Adams^24^ and primarily involves the use of suturing techniques and cephalic trim of the lower lateral cartilages. A cephalic trim is also useful for repositioning malpositioned alar cartilages, but if this technique is not adequate, lateral crural strut or alar contour grafts can be added for further tip support and control.^25,26^

Thin-skinned patients usually require no tip grafts or invisible grafts. Men should not have any supra-tip break, but Middle Eastern women can have a slight supratip break. Any notching in the soft triangle can be corrected with crushed cartilage grafts to help close dead space and soften the appearance of the tip. Be wary of patients with a short-nostril deformity, which may be exacerbated with tip modification techniques. These patients may require soft triangle excision or tip suturing techniques to elevate the nostrils.^27^

If a hanging columella is present, the deformity can be corrected depending on the cause.^28^ For short upper lips and hyperdynamic depressor septi nasi muscles, the muscle can be released to correct this deformity.^6^ Any anterior nasal spine excess can be addressed with fracture and removal of the excess.

## ALAR WIDTH

**Fig. 4.**
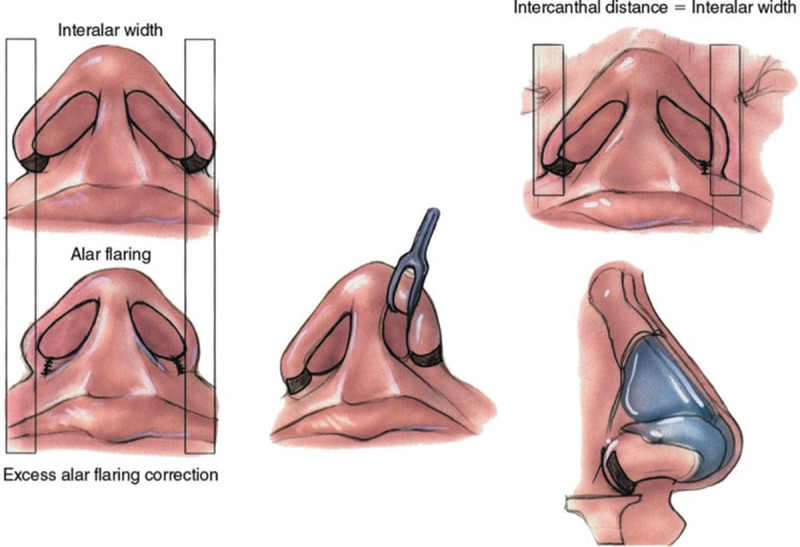
Alar flaring and increased interalar width are common and addressed with alar base surgery. This technique helps address flaring, wide nasal bases, large alae, and nostril asymmetry. *(From Dallas Rhinoplasty 3^rd^ Edition, Eds: Rod J. Rohrich, William P. Adams Jr., Jamil Ahmad, and Jack P. Gunter. 2014, Boca Raton: CRC Press. Reprinted with Permission*).

Alar flaring and increased inter-alar width are common among Middle Eastern patients. If alar flaring is greater than 2 mm from the medial canthus, then alar base excision is recommended (Fig. 4). If inter-alar width is increased, a sill excision can be performed also. Wide alar bases or sills should be reduced in size.^23^ The alar and sill widths should be not be excessively reduced and slight alar flaring is acceptable in Middle Eastern patients. It is better to err on the side of caution and not perform alar base resection in borderline situations with Middle Eastern patients. Any alar malposition can be corrected by reorienting the lower lateral cartilages.^6^

## GOALS IN MIDDLE EASTERN RHINOPLASTY

In Middle Eastern patients, the major goals are creating a stronger internal framework and using visible grafts to create intended effects since the skin and soft tissue is generally thicker. Male Middle Eastern patients require more definition and desire correction of their dorsal hump and tip deformities. These patients do not desire an oversculpted look and do not want to lose a sense of their identity. Female Middle Eastern patients desire tip refinement and a supratrip break.^14^ In Iran, a woman’s nose is exposed and any nasal deformities are linked to her perception of her own attractiveness.^29^ Alar flaring and alar notching are quite common in both genders and can be addressed in primary rhinoplasty with the use of alar contour grafts and tip grafts.^21,24,30,31^

## PITFALLS IN MIDDLE EASTERN RHINOPLASTY

Overcorrection with any of the rhinoplasty techniques can produce an oversculpted appearance, which is not congruent with the Middle Eastern identity.^10^ Tell-tale signs of an oversculpted look include a pinched tip, inverted V deformity, narrow dorsal aesthetic lines, and an overrotated tip. These deformities can occur from excess removal of lower lateral cartilages or excess tip grafting. Racial incongruity can be prevented by performing modest correction of the nasolabial angle and not creating a prominent supratip break, which are features that might be desired in other ethnicities. Especially in patients with thicker skin or more ethnic features, the dorsum should not be overly reduced and the tip should not be overly narrowed.

Pollybeak deformity occurs due to overresection of the caudal dorsum, inadequate resection of the dorsal septum, inadequate cephalic trim, or poor tip projection.^32^ The most common cause in Middle Eastern rhinoplasty is from overresection. In patients with thicker skin, any residual swelling or scarring in the tip can also result in a pollybeak deformity.^32^ Triamcinolone can be injected postoperatively if a pollybeak deformity is suspected to result. Excising soft tissue from the tip to decrease thickness during the primary rhinoplasty has not been shown to prevent pollybeak deformity.^32^

On the other hand, undercorrected patients tend to exhibit the common features found in Middle Eastern patients such as plunging tip and dorsal hump. These patients can also present with a bulbous tip, alar flaring, or internal valve collapse. Alar deformities or collapse can be prevented with the judicious use of alar contour grafts for alar rim support.^31^ Many of these characteristics are found in Middle Eastern patients desiring to undergo primary rhinoplasty (Table 2). Middle Eastern patients desiring revision rhinoplasty tend to have the deformities shown^3,33^ (Table 4).

**Table 4.**
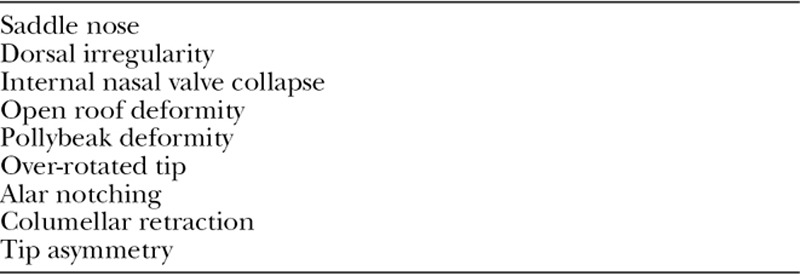
Features in Middle Eastern Patients Desiring Revision Rhinoplasty

## CASE ANALYSES

### Case 1

**Fig. 5.**
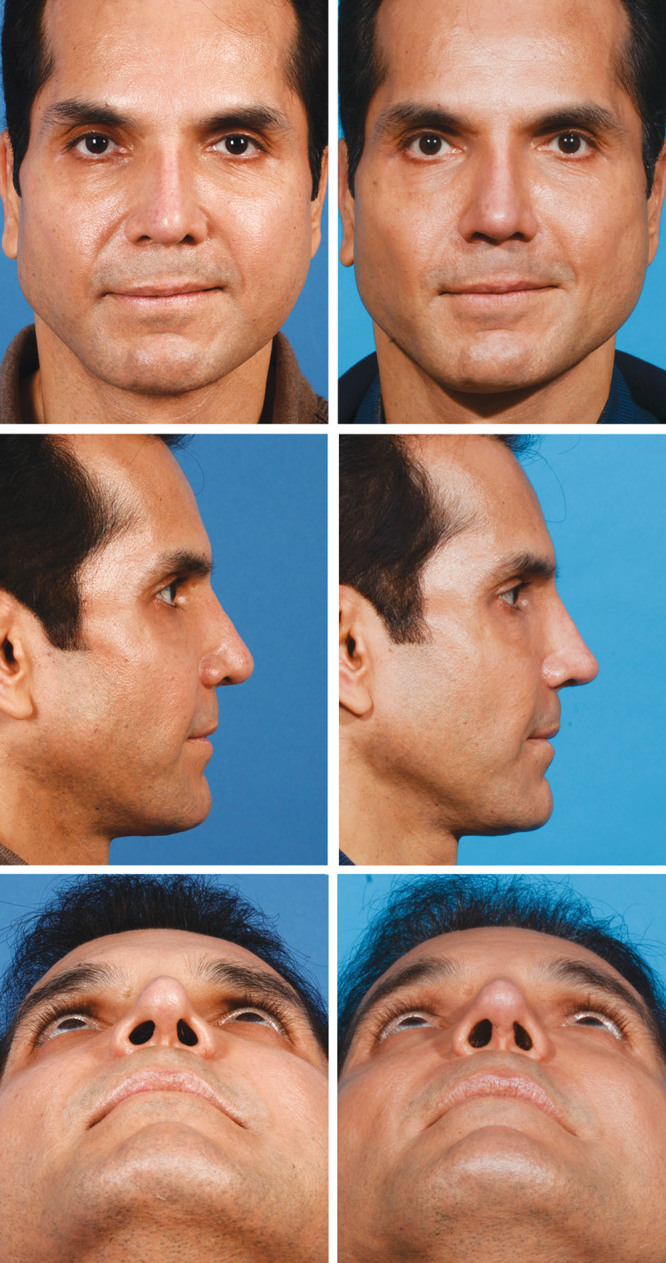
The patient presented with alar rim notching, tip asymmetry, and nasal deviation. He underwent tertiary rhinoplasty using an open approach. The goals were to straighten the nose and correct the asymmetries of the tip.

**Fig. 6.**
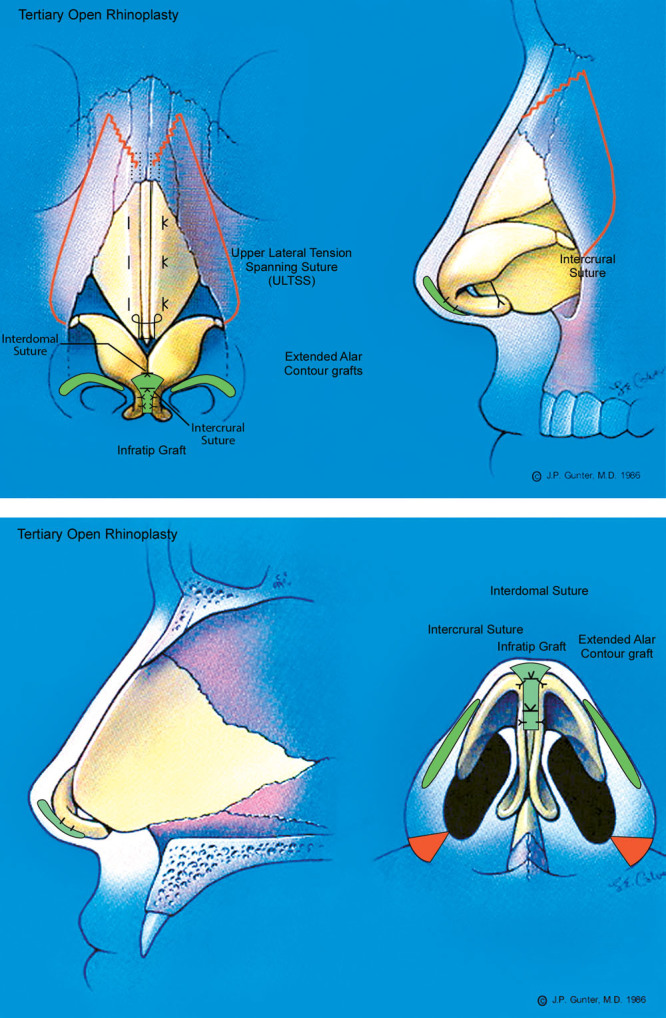
The patient underwent open rhinoplasty with bilateral percutaneous osteotomies followed by maneuvers to improve and refine the tip: tip suturing (intercrural and interdomal sutures), alar base resection, butterfly tip graft, and extended alar contour grafts.

This patient (Fig. 5) underwent tertiary rhinoplasty to correct a deviated nose, wide alar base, and alar notching that developed after a prior rhinoplasty. Through an open rhinoplasty approach, bilateral percutaneous osteotomies and tip refinement were performed using tip suturing (intercrural and interdomal), extended alar contour grafts, and alar base resection (Fig. 6). The osteotomies addressed the deviation; alar base resection reduced the alar width; and the alar contour grafts corrected the alar notching. The patient is shown 2 years postoperatively with better refinement of his ala and a straighter nose.

### Case 2

**Fig. 7.**
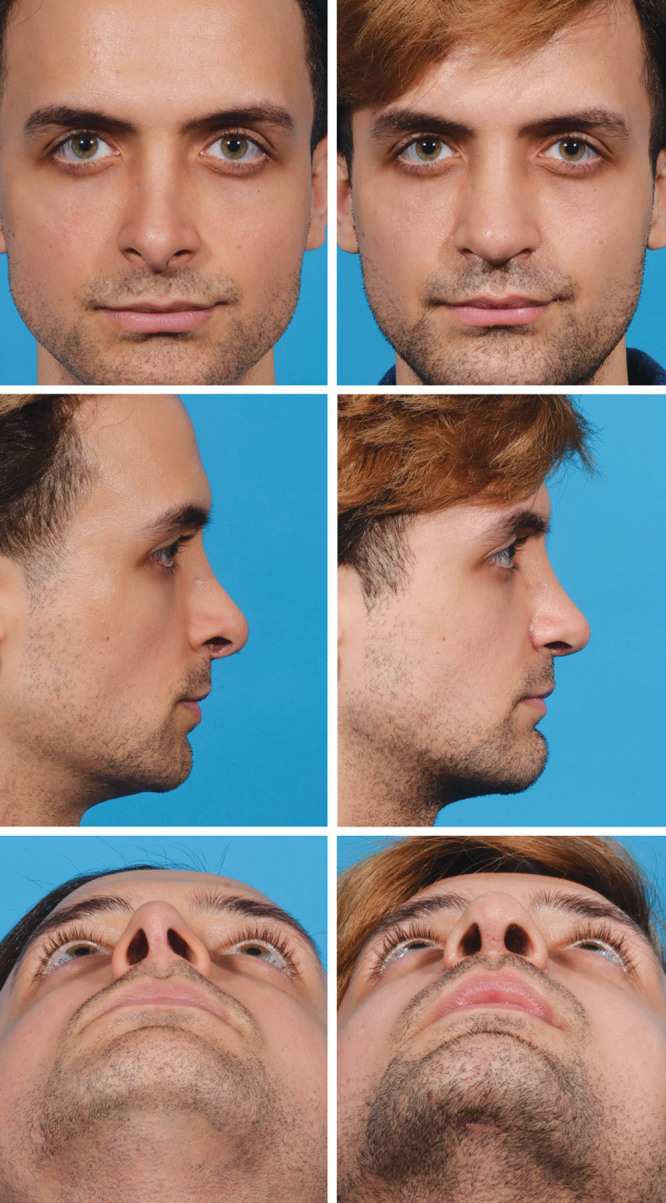
This patient desired tertiary rhinoplasty and chin augmentation to address internal nasal airway breathing problems and aesthetic correction of his tip. An open rhinoplasty was performed using fresh frozen allograft since the patient did not have adequate autologous cartilage for grafting. The patient experienced significant improvement in his breathing and correction of his tip deformities.

**Fig. 8.**
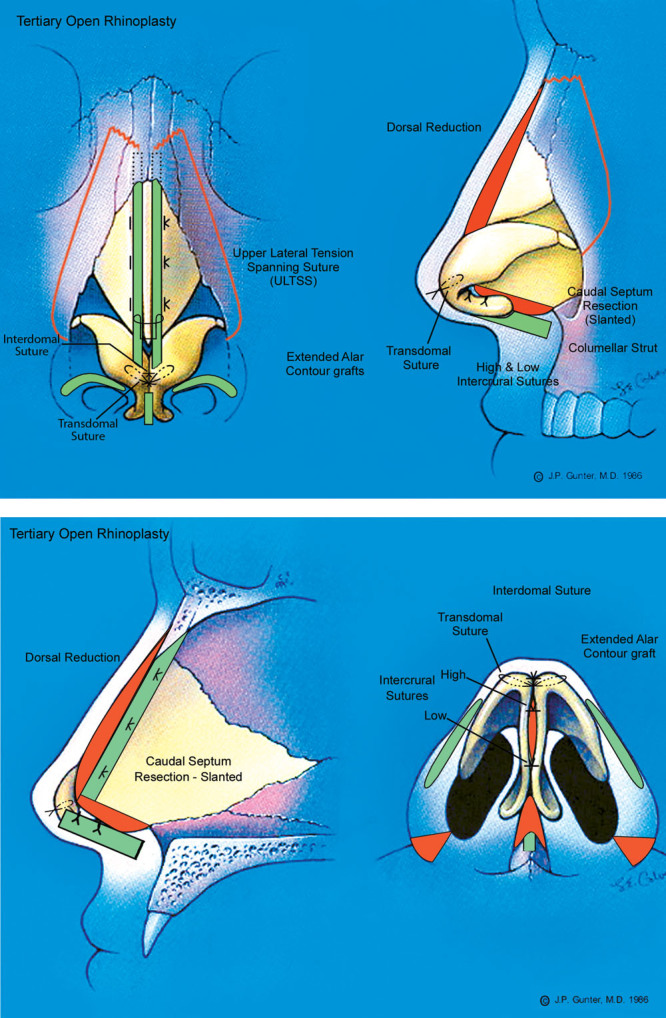
The patient underwent open rhinoplasty with component dorsal reduction, septal reconstruction with caudal septal resection, and bilateral dorsal spreader graft placement using fresh, frozen allograft cartilage. The tip was supported and refined with a columellar strut, interdomal and transdomal sutures, butterfly graft, extended alar contour grafts, and alar base resection.

This patient (Fig. 7) underwent tertiary rhinoplasty and chin augmentation to correct aesthetic and breathing deformities and improve the relationship of the nose and the chin. A component dorsal reduction, septal reconstruction with caudal septal deviation correction, tip suturing (intercrural and interdomal) and grafting (butterfly domal graft), and percutaneous osteotomies were performed (Fig. 8). Extended alar contour grafts were placed and alar base resection was also performed. The patient is shown 6 months after his revision rhinoplasty with a straighter nose and correction of his hanging columella and alar notching. The infra-tip lobular excess and tip asymmetry are corrected and there is residual tip swelling noted. He has a more balanced relationship of his nose and chin.
